# Pediatric Chronic Critical Illness: Protocol for a Scoping Review

**DOI:** 10.2196/30582

**Published:** 2021-10-01

**Authors:** David Zorko, James Dayre McNally, Bram Rochwerg, Neethi Pinto, Rachel Couban, Katie O’Hearn, Karen Choong

**Affiliations:** 1 Department of Pediatrics McMaster University Hamilton, ON Canada; 2 Children’s Hospital of Eastern Ontario Research Institute Children’s Hospital of Eastern Ontario Ottawa, ON Canada; 3 Department of Pediatrics Children's Hospital of Eastern Ontario Ottawa, ON Canada; 4 Department of Medicine McMaster University Hamilton, ON Canada; 5 Department of Health Research Methods, Evidence, and Impact McMaster University Hamilton, ON Canada; 6 Department of Critical Care McMaster University Hamilton, ON Canada; 7 Department of Anesthesiology and Critical Care Medicine Children’s Hospital of Philadelphia Philadelphia, PA United States; 8 Department of Anesthesia McMaster University Hamilton, ON Canada

**Keywords:** pediatrics, critical care, intensive care units, chronic critical illness, research design

## Abstract

**Background:**

Improvements in the delivery of intensive care have increased survival among even the most critically ill children, thereby leading to a growing number of children with chronic complex medical conditions in the pediatric intensive care unit (PICU). Some of these children are at a significant risk of recurrent and prolonged critical illness, with higher morbidity and mortality, making them a unique population described as having chronic critical illness (CCI). To date, pediatric CCI has been understudied and lacks an accepted consensus case definition.

**Objective:**

This study aims to describe the protocol and methodology used to perform a scoping review that will describe how pediatric CCI has been defined in the literature, including the concept of prolonged PICU admission and the methodologies used to develop any existing definitions. It also aims to describe patient characteristics and outcomes evaluated in the included studies.

**Methods:**

We will search four electronic databases for studies that evaluated children admitted to any PICU identified with CCI. We will also search for studies describing prolonged PICU admission, as this concept is related to pediatric CCI. Furthermore, we will develop a hybrid crowdsourcing and machine learning (ML) methodology to complete citation screening. Screening and data abstraction will be performed by 2 reviewers independently and in duplicate. Data abstraction will include the details of population definitions, demographic and clinical characteristics of children with CCI, and evaluated outcomes.

**Results:**

The database search, crowd reviewer recruitment, and ML algorithm development began in March 2021. Citation screening and data abstraction were completed in April 2021. Final data verification is ongoing, with analysis and results anticipated to be completed by fall 2021.

**Conclusions:**

This scoping review will describe the existing or suggested definitions of pediatric CCI and important demographic and clinical characteristics of patients to whom these definitions have been applied. This review’s results will help inform the development of a consensus case definition for pediatric CCI and set a priority agenda for future research. We will use and demonstrate the validity of crowdsourcing and ML methodologies for improving the efficiency of large scoping reviews.

**International Registered Report Identifier (IRRID):**

DERR1-10.2196/30582

## Introduction

### Background

Over the past two decades, the increased survival of even the most critically ill children is greatly attributed to the improvements in the delivery of intensive care [1]. An unintended consequence of this success has been a shift in the population of patients admitted to the pediatric intensive care unit (PICU), with an increasing number of children with chronic or complex medical conditions and significant long-term morbidities following critical illness [1-4]. There is a growing recognition that a subset of pediatric critical illness survivors experience persistent multiorgan system dysfunction and functional morbidities following critical illness that subsequently render them with either a prolonged need for critical care support as inpatients or dependence on medical technology to be cared for as outpatients [5-8]. These children are increasingly recognized as a uniquely high-risk PICU population, also referred to as children with chronic critical illness (CCI) [4,6].

Despite being a uniquely high-risk population in the PICU, research on pediatric CCI remains limited. This patient population has been understudied, largely because of the lack of an accepted consensus case definition. The limited research to date, using variable definitions, suggests that the prevalence of children with CCI is increasing [1,2] and that these children have relatively higher morbidity and mortality rates after critical illness [6,7,9]. These convergent and complex issues exert significant strain on the health care system, health care providers, and caregivers [10-12]. To position the field of pediatric CCI research for systematically evaluating this important patient population, a consistent approach is needed with respect to the population that is being described and studied. Only then is it possible to determine modifiable risk factors for poor patient outcomes, and develop and evaluate interventions to improve the care and survivorship of this important PICU patient population.

### Objectives

Given that we expect a heterogeneous and complex body of work, we have used a scoping review methodology to explore and describe the nature of pediatric CCI research [13,14]. Our primary aim is to evaluate how pediatric CCI is defined in the literature, including concepts such as prolonged or long-stay PICU admission, as it has been proposed that prolonged PICU admissions are important qualifiers for pediatric CCI [4,6]. The secondary aims of this scoping review are to describe the methodologies used to develop and validate any existing definitions of pediatric CCI. We will also seek to describe the prevalence of CCI in the PICU based on existing definitions and describe the key demographic and clinical characteristics of the patient populations studied. Finally, we describe the nature of the reported outcomes in children with CCI.

## Methods

### Protocol

This is an original scoping review following the standard methodology proposed by Arksey and O’Malley [15] and elaborated upon others [13,16]. This protocol is reported in accordance with the Preferred Reporting Items for Systematic Reviews and Meta-Analyses extension for scoping reviews [17]. We uploaded the protocol as a preprint to the Open Science Framework on February 1, 2021 [18]. We plan to document protocol amendments in the Open Science Framework with the date, description, and rationale. Patients and the public were not involved in the design, conduct, reporting, or dissemination plans of this research.

### Eligibility Criteria

#### Types of Participants or Population

We will include studies that evaluated critically ill children (ie, <18 years old) admitted to any PICU, explicitly identified with *CCI*. We will also include studies that evaluated prolonged, protracted, chronic, or long-stay PICU admission, as this concept has been identified as an important qualifier for pediatric CCI. However, we excluded records if they (1) evaluated adult or neonatal intensive care unit populations only, or included children among these populations but did not report separate data for children; (2) evaluated pediatric patients in intermediate care, step-down, high-dependency, or chronic ventilator or respiratory units; and (3) did not include or reference a definition of pediatric CCI or prolonged PICU admission, as applicable to the study (eg, as a case definition in a prevalence study).

#### Types of Interventions, Comparators, and Outcomes

We will not apply any restrictions regarding interventions, comparators, or outcomes.

#### Types of Publications

We will include observational and experimental studies, qualitative studies, and protocols that provide a working definition of pediatric CCI or prolonged PICU admission. Then, we will exclude literature reviews, unpublished literature, editorials, commentaries and opinion pieces, conference proceedings, abstracts, and books. Given the emerging nature and recognition of CCI in children, we will exclude records published before 1990. We will exclude studies that were not published in English or French.

### Search Strategy

We developed a preliminary search strategy in two electronic databases (MEDLINE and CINAHL) and piloted this in consultation with a health research librarian (RC). We developed the final search strategy in MEDLINE, which was peer-reviewed by 2 additional health research librarians not involved in the study, and then translated it into the other databases, as appropriate (Textbox 1). We will search four databases that index citation titles or abstracts using English Medical Subject Headings terms and keywords from their dates of inception to March 2021: Ovid MEDLINE, Embase, CINAHL, and Web of Science. We will review the reference lists of all included studies to identify any studies that may have avoided the final database search.

**Search strategy (MEDLINE). adj: adjacent; epub: electronic publication; exp: explode; .mp: multi-purpose; PICU: pediatric intensive care unit.**

**Database**

- Ovid MEDLINE epub ahead of print, in-process and other nonindexed citations, Ovid MEDLINE(R) daily and Ovid MEDLINE(R) 1946-present

**Search strategy**

- intensive care units/and (child* or pediatric or paediatric).mp. [mp=title, abstract, original title, name of substance word, subject heading word, floating sub-heading word, keyword heading word, organism supplementary concept word, protocol supplementary concept word, rare disease supplementary concept word, unique identifier, synonyms]
- Intensive care units, pediatric/
- PICU.mp.
- ((p?ediatric* or child or children*) adj3 (acute* or critical* or intens*)).mp.
- or/1-4
- exp Critical Care/
- Critical Illness/
- (critical* or intens*).mp. [mp=title, abstract, original title, name of substance word, subject heading word, floating sub-heading word, keyword heading word, organism supplementary concept word, protocol supplementary concept word, rare disease supplementary concept word, unique identifier, synonyms]
- or/6-8
- exp chronic disease/
- length of stay
  /
- ((long or duration or length) adj3 (stay or hospitali*)).mp. [mp=title, abstract, original title, name of substance word, subject heading word, floating sub-heading word, keyword heading word, organism supplementary concept word, protocol supplementary concept word, rare disease supplementary concept word, unique identifier, synonyms]
- or/10-12
- 5 and 9 and 13
- ((chronic* or persist* or long term or longterm or long-stay or prolong* or protract* or extend* or extensive or lengthy or difficult*) adj5 (acute* or critical* or intens* or ill or illness* or sick or sickness* or care)).mp. [mp=title, abstract, original title, name of substance word, subject heading word, floating sub-heading word, keyword heading word, organism supplementary concept word, protocol supplementary concept word, rare disease supplementary concept word, unique identifier, synonyms]
- 5 and 15
- 14 or 16
- ((p?ediatric* or child or children*) adj5 (chronic* or persist* or long term or longterm or prolong* or protract* or extend* or extensive or lengthy or difficult* or ((long or duration) adj3 stay)) adj5 (acute* or critical* or intens* or ill or illness* or sick or sickness* or care)).mp.
- 17 or 18

### Study Selection

#### Search Strategy and Study Selection Criteria Piloting

The team used an iterative approach to evaluate and refine the preliminary search strategy and study selection criteria. Using the results of the preliminary search strategy, 4 members of the core study team independently reviewed an initial set of 100 randomly selected citations using the initial study selection criteria. Each record was reviewed in triplicate. We screened the 100 citations in two steps (title and abstract, then full text), discussed discrepancies, and refined the eligibility criteria. The lead investigator (DZ) reviewed the reference lists of studies meeting all inclusion criteria, identified any relevant studies, and, together with the health sciences librarian, refined the search strategy if these relevant studies were missed by the database search. Following this initial round, we reevaluated the revised study selection criteria using a second set of 100 random citations assessed independently and in triplicate. The conflict rates were 45.5% (5/11 full texts) and 7.7% (1/13 full texts) in full-text assessment during the two iterative piloting rounds. Following these two iterative piloting rounds, the team established a consensus on the study selection criteria. A total of 8 eligible studies were identified during the piloting.

#### Crowdsourcing

Given the large number of citations identified in the final search strategy, we will use a hybrid approach comprising crowdsourcing and a machine learning (ML) algorithm to expedite the screening of records. The crowdsourcing methodology for systematic reviews has been previously validated [19,20] and used in a variety of health research reviews to accelerate the citation screening and provide more timely research output, while still allowing for rigorous review conduct [21-23]. We will recruit a curated crowd of approximately 30 English- and French-speaking reviewers with content and methodological expertise from international PICU networks (eg, Canadian Critical Care Trials Group, Pediatric Acute Lung Injury, and Sepsis Investigators group), email, social media (using the hashtags #PedsICU, #PICSp, and #CCI), and a dedicated study crowdsourcing event page on insightScope [24]. Authorship incentives will be offered to crowd reviewers who achieved specific screening milestones (ie, group authorship if ≥500 abstracts and ≥50 full texts screened, named authorship if ≥1000 abstracts, ≥100 full texts screened, and participated in data abstraction).

Before formal screening, prospective reviewers will be provided with a copy of the protocol and selection criteria. Prospective reviewers will first perform screening on a test set designed using the piloted study selection criteria [25]. The test set will contain 100 citations from the pilot phase with 10 eligible (true positive) citations. Prospective reviewers must achieve a sensitivity of ≥80% before they are given access to the full set of study records. Reviewers who do not achieve ≥80% sensitivity will be provided with additional training before being given access to the full set of study records.

We will use a dedicated channel on Slack (Slack Technologies), a cloud-based team communication platform, to streamline the study progress updates and reviewer communication [26,27].

#### ML Algorithm

**Figure 1:**
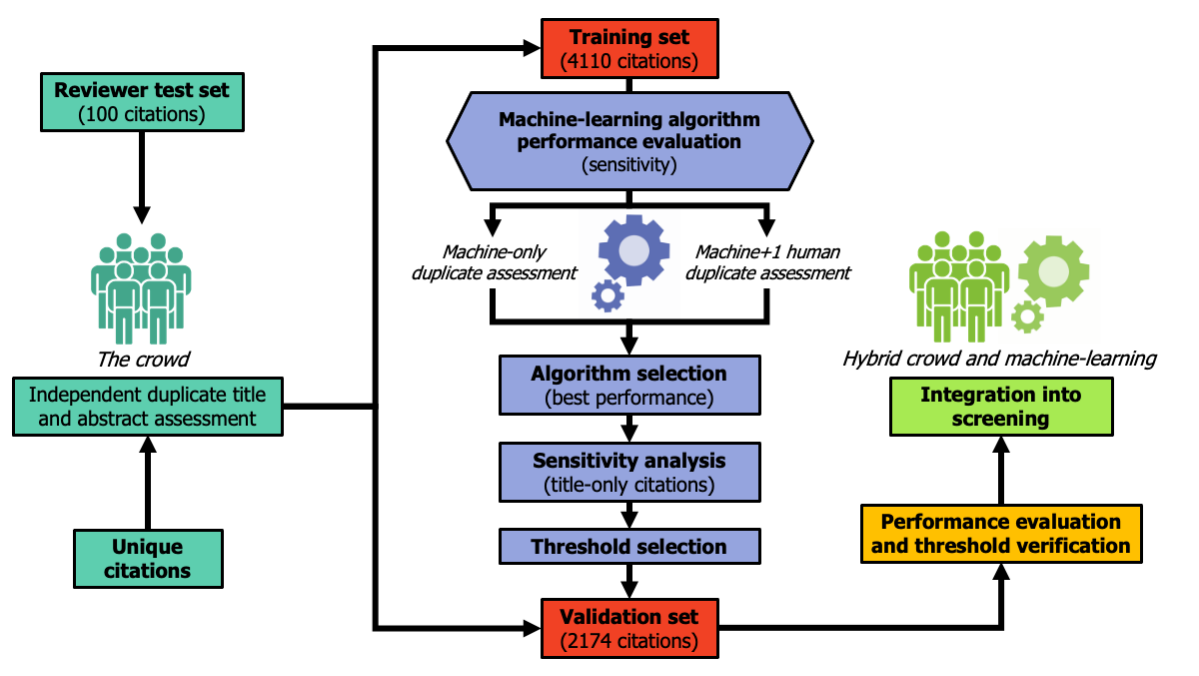
Integration of crowdsourcing and machine learning in the scoping review.

ML algorithms are being increasingly used to assist in citation screening for systematic reviews, particularly in large reviews [28-31]. We will develop an ML algorithm to semiautomate citation screening for this scoping review at the title and abstract stage only, which is consistent with previously described approaches (Figure 1) [31]. The independent and duplicate screening of at least 4000 citations through to the full text by crowd members will constitute a *training set* that we will use to evaluate five ML algorithms (bag of words, term frequency-inverse document frequency, word to vector, document to vector, and fast text). These algorithms assess the citation title and abstract (where available) and rank each citation by relevance based on the text captured in the study selection criteria and project goal, with the highest ranking citations being retained based on a threshold set by the investigator (eg, a threshold of 70% would retain the 30% highest ranking citations). The titles and abstracts of citations from the four electronic databases were downloaded in English; therefore, no language adaptations were required to apply the ML algorithms to non–English-language studies.

We will select the two highest performing algorithms from the training set and evaluate their sensitivity and specificity at a variety of thresholds, when used alone and in combination with a single human reviewer. We will also separately evaluate the performance of the two highest performing ML algorithms for citations without an abstract (ie, title only) to evaluate whether a unique threshold would be required. For both ML algorithms, we will determine the threshold at which the sensitivity is >95% when used in combination with a single human reviewer. This approach is consistent with the individual sensitivity of *expert* reviewers, as described in previous studies [20,23,32,33].

Once developed, we will evaluate the performance of the two candidate ML algorithms on an additional *validation set* constituting at least 2000 citations screened independently in duplicate by crowd members. Our a priori methodology will be to proceed with the duplicate independent human assessment of citations above the selected threshold score, and machine plus one independent human assessment for citations below the threshold score. We will also plan to apply an additional lower threshold score if the sensitivity data for the candidate ML algorithms consistently exceed our sensitivity goal (ie, 95%). This lower threshold will serve to exclude the most irrelevant citations through assessment by the ML algorithm alone.

#### Integration of Hybrid Crowdsourcing and ML Algorithm Citation Screening

The integration of crowdsourcing and ML algorithm methods into citation screening in this scoping review is outlined in Figure 1. We will download records from the electronic search into Endnote for duplicate removal and export the citation list for screening to insightScope [34], a platform for executing large reviews through crowdsourcing. We will upload citation abstracts and full-text articles with inclusion and exclusion criteria for insightScope. Screening will be performed in two steps (title and abstract, then full text) against the inclusion criteria by 2 independent reviewers. We will record reasons for the exclusion of citations excluded from full-text screening. As previously described, no language adaptations to the screening process for non-English studies will be required for the title and abstract stage, as citations retrieved from electronic databases are in English. However, full texts in French will be reviewed independently and in duplicate by French-speaking crowd reviewers. All screening conflicts (either between 2 humans or a machine and 1 human) will be resolved by third-party adjudication by the members of the core study team, as required.

### Data Charting

We will perform data abstraction using the piloted electronic data abstraction forms created in insightScope. The data abstraction forms were created by one investigator (DZ) and piloted by the members of the core investigative team (JDM, BR, NP, KO, and KC) against a total of 8 eligible studies. We have described the data items in Textbox 2. Before formal data abstraction, we will provide all data abstractors with training (ie, a data abstraction manual and training video). Data will be abstracted by 2 independent reviewers from the crowd, both independently and in duplicate. We will abstract data from the full-text publication and any related publications, referenced published protocols, or supplementary materials. Where necessary, one reviewer will extract graphical data using SourceForge Plot Digitizer, which will be checked by the second reviewer for accuracy. Moreover, where necessary, data will be abstracted from publications in French by French-speaking crowd reviewers independently and in duplicate. The study lead (DZ) resolved conflicts in data abstraction, as required. In the event of missing or unclear data related to our outcomes of interest, we will make a maximum of three attempts to contact the study authors for clarification.

**Data items.**

**Study characteristics**

- Author name and contact information
- Title
- Country of origin
- Journal and year of publication
- Study design
- Clinical setting and type of pediatric intensive care unit (eg, medical-surgical, cardiac only, and neuro-pediatric intensive care unit)
- Participant inclusion and exclusion criteria
- Total patients included
- Study period (dates)

**Study population definition**

- Definition of pediatric chronic critical illness (eg, as defined by study or referenced from another publication)
- Definition of prolonged pediatric intensive care unit or long-stay admission (eg, duration, as defined by study or referenced from another publication)
- If and how the definition was developed or validated by the primary study
- Prevalence of study participants with chronic critical illness or prolonged pediatric intensive care unit admission, as applicable to the study

**Study population demographics and characteristics**

- Age and sex
- Reason for pediatric intensive care unit admission
- Source of pediatric intensive care unit admission (eg, emergency department, neonatal intensive care unit, floor or step-down unit)
- Functional status characteristics (using validated tools, as categorized by the article)
- Severity of illness characteristics (using validated tools, as categorized by the article)
- Comorbidity and medical complexity status, including if and how patient medical complexity and comorbidity was described in the study
- Prevalence and types of organ support technologies in study participants (eg, mechanical ventilation, feeding support, circulatory support [vasoactive drugs, extracorporeal membrane oxygenation, ventricular assist device], and extrarenal filtration)
- Types of study participants (eg, children with chronic critical illness or prolonged pediatric intensive care unit admission, families, siblings, and health care providers)

**Outcomes evaluated**

- Stated primary outcome, including how it was measured and result
- Patient outcomes, including mortality (pediatric intensive care unit, hospital, and overall), discharge disposition (eg, high-dependency unit, ward, rehabilitation facility, and home), and health-related quality of life
- Family and sibling outcomes (any, as categorized by the article)
- Health care provider outcomes (any, as categorized by the article)
- Health care system outcomes, including the length of stay (pediatric intensive care unit and hospital), pediatric intensive care unit bed-day use or consumption, pediatric intensive care unit readmission rate or occurrence, and pediatric intensive care unit cost analyses

### Results Synthesis

We will report data related to study characteristics descriptively using counts with the percentages or measures of central tendency and variance (eg, means/medians with SDs/IQR), as appropriate. We will use tables to narratively summarize data related to study population definitions, including the prevalence of the population studied (if applicable) and contextual variables related to study type, setting, and evaluated patient population. We will describe the important elements of the methodology used to derive the case definition of CCI and prolonged PICU admission, including but not limited to the size of study, study design, setting(s), and if criteria for agreement or convergence established a priori. We will group included studies into one of the two definition domains based on their explicitly identified study population of interest (ie, CCI or prolonged PICU admission) and summarize data for each, separately. We plan to categorize patient- and family-based outcomes evaluated in the included studies according to the domains of the PICU Core Outcome Set [35] (ie, overall health, cognitive function, physical function, and emotional function), as applicable, to help formulate a priority agenda for future research.

Statistical analyses will be performed using SPSS Statistics, version 26 (IBM), as necessary. We will not perform any meta-analyses of epidemiological or outcome data collected from primary publications, in keeping with the descriptive nature of this scoping review. In keeping with a scoping review methodology, we will not complete the risk of bias assessment for included studies or undertake the certainty of evidence assessment for this scoping review [13,14]. However, the limitations of the nature and extent of populations and outcomes evaluated in current pediatric CCI research will be addressed in the Discussion section of the paper.

## Results

Database search, citation screening, and the data abstraction phases of this scoping review started on March 3, 2021, and were completed on April 16, 2021. Data verification is ongoing, with data analysis as follows: the analysis of the review, with results, is anticipated to be completed by fall 2021.

## Discussion

### Crowdsourcing and ML Algorithm Methods

A total of 32 crowdsourced reviewers completed the test set of 100 citations, achieving a mean sensitivity of 91.6% (SD 0.09). Two reviewers with exactly 70% sensitivity on the test set were provided additional training on the study protocol and study selection criteria before citation screening. Of these, 28 reviewers, with a test set sensitivity of 92.1% (SD 0.09), participated in the citation screening. Reviewers originated from 11 countries and 5 continents.

**Figure 2:**
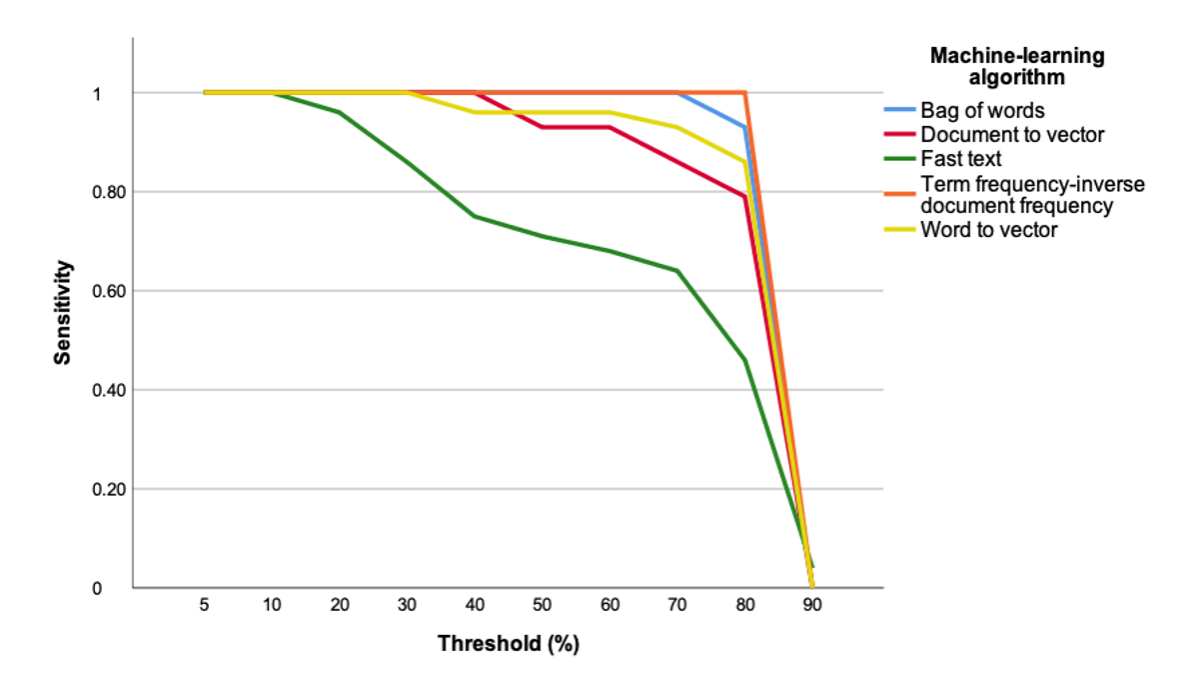
Machine learning algorithm training set performance (machine-only citation assessment). The bag-of-words and term frequency-inverse document frequency demonstrate the highest sensitivities up to a threshold of 80%.

**Figure 3:**
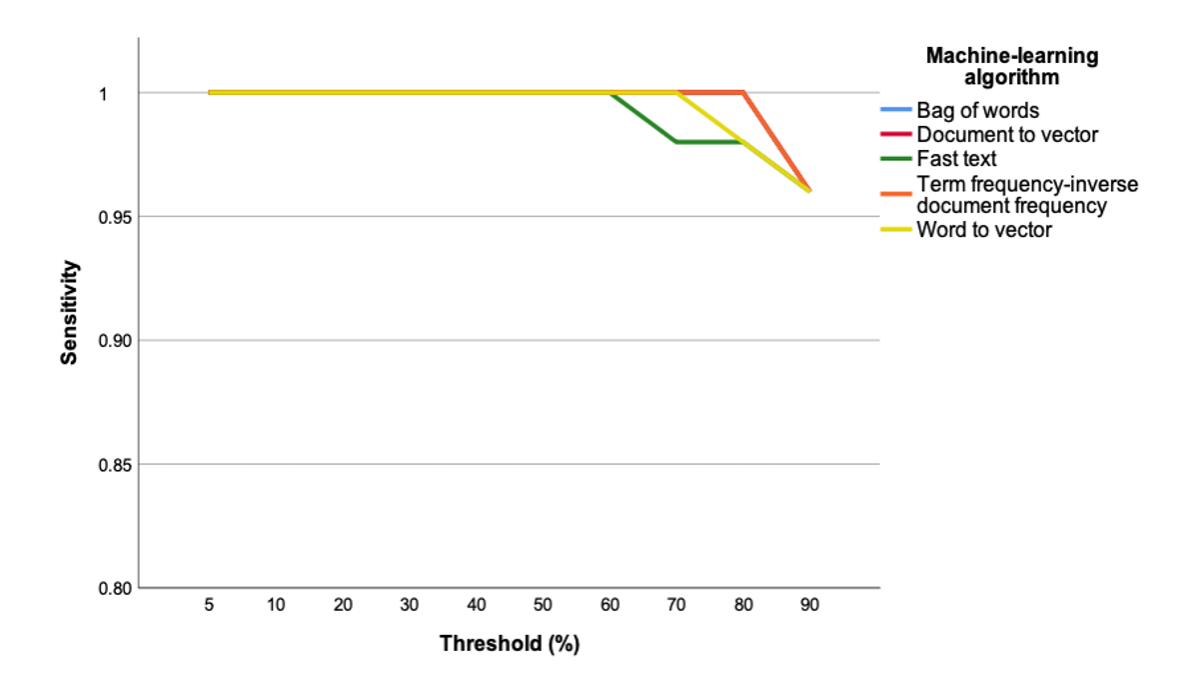
Machine learning algorithm training set performance (machine+one human reviewer citation assessment). The document to vector line overlaps with term frequency-inverse document frequency. The bag-of-words line overlaps with term frequency-inverse document frequency, demonstrating a sensitivity of 100% at a threshold of 80%.

As a prerequisite to incorporate an ML algorithm into citation screening, we determined the optimal algorithm and sensitivity threshold for operationalization. The sensitivities of the five evaluated ML algorithms when used alone or in combination with a single human reviewer to assess citations from the training set are presented in Figures 2 and 3, respectively. The 4110-citation training set included 28 citations meeting the inclusion criteria following an assessment by 2 reviewers after full-text review (ie, true positives). The two highest performing ML algorithms were bag of words and term frequency-inverse document frequency, demonstrating 93% and 100% sensitivity, respectively, at a threshold of 80% when citation assessments were performed by the ML algorithm alone. The sensitivities for both these ML algorithms were 100% at a threshold of 80% when citation assessments were performed by the ML algorithm in combination with a single human reviewer.

Additional sensitivity analyses were performed using the bag of words and term frequency-inverse document frequency algorithms using a separate threshold for citations without an abstract (ie, title only) to evaluate whether these citations perform differently. For this analysis, the threshold for citations with an abstract was fixed at 70%, and the threshold for citations without an abstract varied among 30%, 50%, and 70%. The bag of words and term frequency-inverse document frequency algorithms demonstrated sensitivities of 100% for all dual threshold combinations (ie, 70/30, 70/50, and 70/70), both when citations were assessed by the ML algorithm alone or in combination with a single human reviewer.

We subsequently evaluated the bag of words and term frequency-inverse document frequency ML algorithms on a validation set of 2174 additional citations. Again, these citations were screened independently and in duplicate by crowd reviewers. The validation set included nine unique citations that met the inclusion criteria. On the basis of the sensitivity results from the training set, we chose to apply the following conservative thresholds to evaluate performance on the validation set: 70% for citations with an abstract and 50% for citations with title only. Both the bag of words and term frequency-inverse document frequency algorithms demonstrated a sensitivity of 92% when citations were assessed using the ML algorithm alone, and a sensitivity of 100% when used in combination with a single human reviewer.

In addition to sensitivity, we evaluated the specificity of the ML algorithm. Both the term frequency-inverse document frequency and bag of words algorithms demonstrated a similar specificity at 70% threshold (ie, 0.68), but the term frequency-inverse document frequency algorithm retained three fewer false positive citations. Given this marginally better performance, term frequency-inverse document frequency was selected as the final ML algorithm. Considering that ML algorithms are relatively novel in the conduct of large scoping reviews, we adopted a conservative approach to integrating the algorithm into citation screening for the remaining citations in the review. For citations with an abstract, the following three thresholds were selected:

1. Citations with a score ≥70% threshold were assessed by duplicate independent human assessment.
2. Citations with a score between 30% and 70% threshold were assessed by machine plus one independent human assessment.
3. Citations with a score ≤30% threshold were assessed by machine-only assessment.

For citations without an abstract (ie, title only), we adopted a conservative approach by selecting a 50% threshold and no option for machine-only citation assessment. Therefore, citations with a score ≥50% threshold were assessed by duplicate independent human assessment, and citations with a score <50% threshold were assessed by machine plus one independent human assessment.

### Strengths and Limitations

This scoping review is the first phase of a larger research program to systematically evaluate children with CCI. To our knowledge, this scoping review is the first evidence synthesis to provide a systematic overview of the definitions used in the literature for identifying children with CCI and prolonged PICU admission. As such, the results of this review will be used to inform the development of a consensus case definition for pediatric CCI and set a priority agenda for future research. Defining pediatric CCI is an essential first step in understanding the epidemiology of this high-risk PICU population, and a prerequisite for conducting future interventional and outcomes research. As the aims of this scoping review are descriptive and exploratory in nature, this preliminary study will identify the potential need to conduct a systematic review to address targeted and explanatory epidemiologic questions. This scoping review will also demonstrate the feasibility and validity of two innovative evidence synthesis methods, crowdsourcing and an ML algorithm, to execute a large scoping review.

This review has several important limitations. As the goal of this scoping review was to describe the definitions of pediatric CCI and prolonged PICU admission, it is limited to studies that explicitly identified and defined these concepts. This review will potentially miss records that did not use this specific language to define their population, and excluded studies that did not provide or reference a definition of pediatric CCI or prolonged PICU admission. Similarly, the study selection criteria in this review will exclude studies that focused only on the concept of prolonged technology use (eg, prolonged mechanical ventilation, prolonged extracorporeal membrane oxygenation). We seek to broadly understand pediatric CCI, and as a part of this objective, we will describe how the concept of organ support technology is applied in the published definitions of pediatric CCI.

### Conclusions

This scoping review is the first, to the best of our knowledge, to (1) provide a systematic overview of the definitions used in the literature for identifying children with CCI and prolonged PICU admission and (2) describe the demographic and clinical characteristics of the populations historically defined in the pediatric CCI literature. This comprehensive literature review will evaluate existing or suggested definitions of pediatric CCI. In the absence of definitions, the review results will be used in future research to identify the key terms and constructs to inform the development of a working definition of pediatric CCI. Defining pediatric CCI is an essential first step in understanding the epidemiology of this high-risk PICU population and a prerequisite for conducting future interventional and outcomes research.

## Abbreviations

CCI: chronic critical illness
ML: machine learning
PICU: pediatric intensive care unit
